# What drives perception of decline in seafood culture?: The mediating role of memory in local coastal cuisine in Japan

**DOI:** 10.1016/j.crfs.2026.101482

**Published:** 2026-06-24

**Authors:** Yining Wang, Ryo Kohsaka

**Affiliations:** Graduate School of Agricultural and Life Sciences, The University of Tokyo, Tokyo, Japan

**Keywords:** Seafood consumption, Dietary habits, Food-related memory, Cultural identity, Food culture decline, Mediation analysis

## Abstract

Amid globalization and accompanying shifts in dietary habits, local food cultures continue to decline in both consumption quantity and quality. This study examines the relationships among seafood consumption habits, food-related memory, and perceptions of the decline in traditional local cuisine, focusing on both at-home and out-of-home dining contexts. Integrating experiential memory theory with the concept of declining locality, we propose a “habit–memory–framing” framework to examine the interplay among dietary habits, food-related memory, and cultural perception. Drawing on online survey data from 840 respondents, we conduct multiple regression and mediation analyses to investigate the underlying mechanisms. The results indicate that seafood consumption habits indirectly influence perceptions of cultural decline via food-related memory while exerting a direct negative effect on perceptions of decline in seafood culture, forming a dual-path structure. This implies that dietary habits attenuate the direct perception of cultural decline at the behavioral level and enhance individuals’ sensitivity to cultural change through memory-based cognitive mechanisms. Comparisons between at-home dining and out-of-home dining contexts reveal that the home setting (or at-home dining) plays a more prominent role in shaping food-related memory than out-of-home dining. Further analysis identifies price, time constraints, and product availability as factors affecting seafood consumption habits. These findings suggest that changes in local food culture are shaped not only by behavioral patterns but also by emotional experiences and cognitive mechanisms. By integrating consumption habits and food-related memory, this study provides new empirical insights into the dynamics of food culture change, offering implications for policy and practice.

## Highlights

• Proposes a “habit–memory–framing” framework linking consumption and cultural perception.
• Identifies a dual-path mechanism: direct and memory-mediated effects of consumption habits.
• Demonstrates the key mediating role of food-related memory in perceived cultural decline.
• Shows that at-home dining more strongly shapes food-related memory than eating out.
• Reveals price, time, and availability as constraints on seafood consumption.

## Graphical abstract

**Figure:**
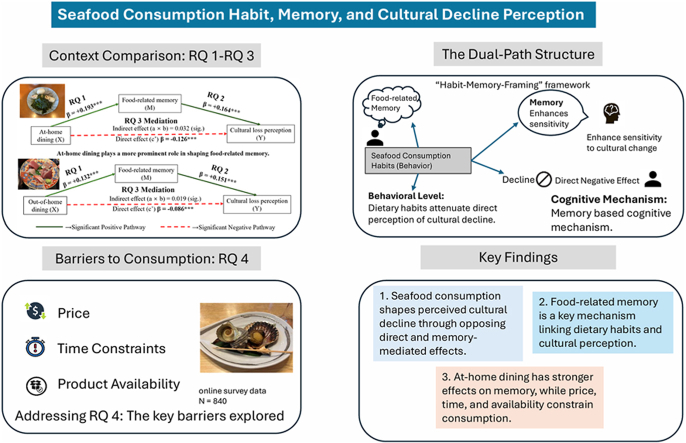

## 1 Introduction

In recent years, the relationship between dietary behavior and cultural framing has garnered scholarly attention. Local cuisines and regional food traditions are widely regarded as important forms of cultural heritage that play a central role in cultural transmission (Lee, 2023). Rather than merely fulfilling a physiological need, food consumption constitutes a key site for meaning-making, where the continuous reproduction of practice and memory sustains intangible cultural heritage (Bortolotto, 2015). Everyday food practices actively embed individuals within broader social structures and reflect lifestyle choices (Batat et al., 2019; Johnston, 2018), and food is a social construct in tourist destinations for tourist “gaze” (Kim et al., 2010; Kovalenko et al., 2023). Through emotional engagement, these daily food experiences enhance individuals’ identification with places and strengthen community attachment (Bonn, 2015; Sthapit, 2017; Moreno and Malone, 2021). Collectively, these studies demonstrate that local cuisine functions as a dynamic medium through which cultural resonance and identity are actively generated (Yu et al., 2021). Seafood in coastal areas is no exception; it frequently plays a central role in the meaning creation of destinations (Banerjee and Quinn, 2022).

**Fig. 1:**
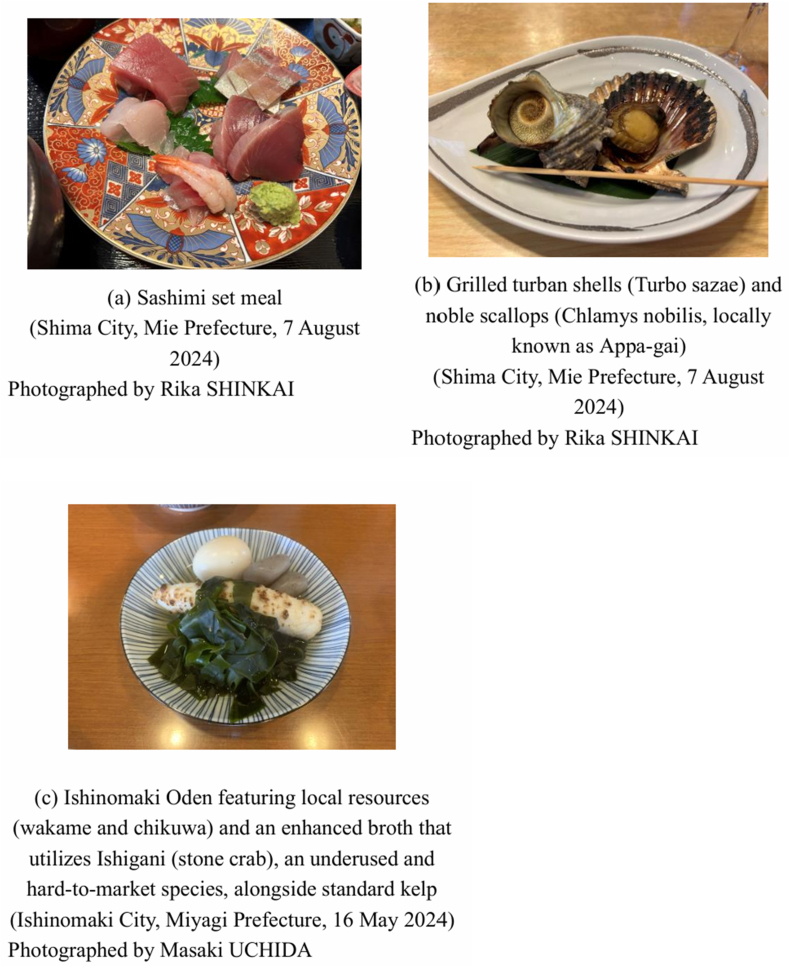
Examples of changing marine foodscapes in Japanese coastal areas.

While such cultural embeddedness is crucial, it is increasingly threatened by various factors, such as rapid changes in social norms, pandemics, and global environmental changes, including climate changes that impact both terrestrial and coastal areas. The expansion of ultra-processed food is actively reshaping traditional dietary patterns (Monteiro et al., 2018). Globalized food systems risk weakening the cultural roots of local food practices (Mak et al., 2012a). More importantly, with the increasing shift of dining contexts from the domestic sphere to external consumption spaces, the situational foundations of traditional food memories are fundamentally being altered (Zhu et al., 2024). This spatial shift subsequently drives an evolution in how individuals perceive local cuisine and its cultural significance (Şahin and Kılıçlar, 2022). In coastal destinations, as the consumption of traditional seafood moves from local households and community fish markets to commercialized dining venues (Souza Olegario et al., 2021), the authentic cultural connection to marine foodscapes is reshaped. Simultaneously, to cope with environmental changes and declining catches, coastal communities are actively adapting their food practices by utilizing previously underrepresented marine resources (Fig. 1).

Prior research has highlighted the mediating role of memory in understanding how shifting norms and practices influence cultural framing. Food acts as a medium for personal experiences and social relationships (Holtzman, 2006; Conway, 2005). Dietary experiences evoke memory associations related to family and region, which gradually accumulate into stable food-related memories (Sutton, 2001). Furthermore, traditional food experiences have profound cultural significance and are closely intertwined with local identity (Bessière, 2013). Related studies have shown that local food consumption can strengthen the connection between individuals and regional cultures (Sims, 2009). This process aligns closely with experiential memory theory, which posits that daily embodied practices, particularly domestic cooking and dining, serve as critical contexts for generating and sustaining deep emotional memories (Dantec et al., 2021; Pan et al., 2025).

Building on this, social cognitive theory posits that individuals' behavioral experiences influence their subsequent attitudes and judgments through experience accumulation and cognitive processing mechanisms (Bandura, 1986). Applying this to the changing food landscape suggests that shifting dietary habits are internalized by changing emotional memories (Kensinger, 2009), which subsequently reshape individuals’ broader cultural evaluations (Chen et al., 2025). Previous research has verified the relationship between local food and cultural identity (Kim et al., 2021), and the association between food experiences and memory construction has been explored (Stone et al., 2019). However, these theoretical discussions have rarely been integrated to address the contemporary crisis in local food culture. Specifically, how the shifting dynamics of everyday food practices—such as the contrast between at-home and out-of-home dining—translate, via emotional memory, into the subjective, micro-level perception that “local cuisine is disappearing” remains unclear. In addition, limited attention has been paid to coastal areas, and the role of traditional seafood in non-Western contexts remains largely unexplored (Cisneros-Montemayor et al., 2016; Ward et al., 2024). Studies on foodscapes in coastal areas that use visual and text materials are also limited (Uchida et al., 2025).

**Fig. 2:**
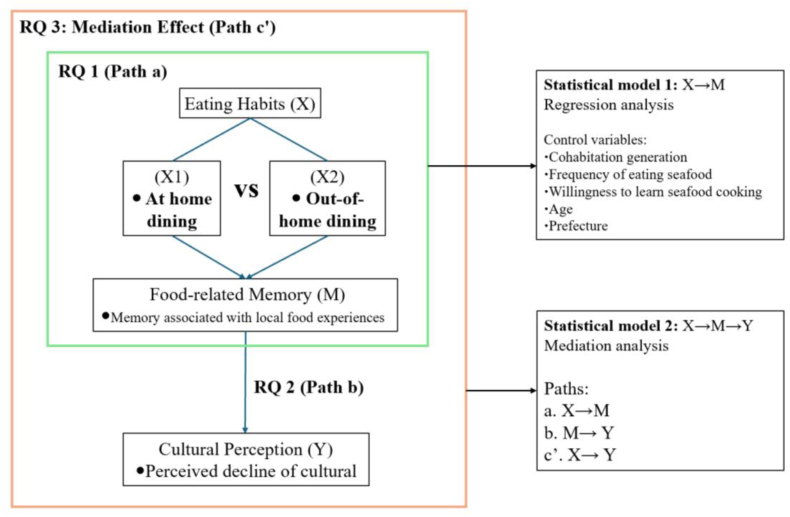
Conceptual framework.

To bridge these gaps in the literature and connect theoretical discussions to empirical realities, we propose a theoretical framework of “habit–memory–framing” that links dietary habits, food-related memory, and cultural perception based on empirical datasets at the individual level. Specifically, using seafood consumption as a representative context for local food culture, we investigate how individuals’ dietary habits influence their perception of the decline in local cuisine through the mediating role of food-related memory (Fig. 2). By controlling for demographic variables such as age, co-residing generations, and place of residence, this study tests the mediating effect of food-related memory between dietary habits and cultural perception. By elucidating this micro-level psychological mechanism, we aim to identify the relationship between dietary practices and cultural framing to gain insights into the preservation of traditional local cuisine in coastal areas.

Based on this framework, we propose four research questions: (RQ1) How do dietary habits influence food-related memory in at-home and out-of-home dining contexts?; (RQ2) How does food-related memory affect cultural perception?; (RQ3) Does food-related memory mediate the relationship between dietary habits and cultural perception?; and (RQ4) What factors contribute to the decline in seafood consumption at home?

## 2 Methodology

### 2.1 Study Area and sample

**Fig. 3:**
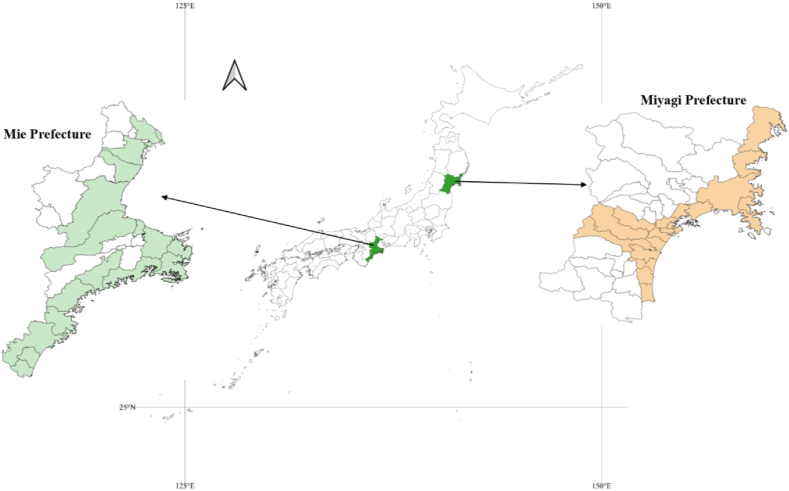
Study area.

This study selected coastal municipalities in two Japanese prefectures, Miyagi (15 municipalities) and Mie (18 municipalities), as research sites (Fig. 3). These areas were chosen for multiple reasons. First, both prefectures have extensive coastlines and active fishing industries, and their local food cultures are closely connected to marine resources, a characteristic also reflected in relevant statistical data. According to prefecture-level fish catch statistics for coastal regions, Miyagi Prefecture recorded a total catch of 164,547 tons, ranking fourth, whereas Mie Prefecture recorded a catch of 584,522 tons, ranking 13th. These figures indicate that both prefectures occupy relatively important positions and are representative of Japan's coastal fishery system (e-Stat, 2024). Second, Miyagi Prefecture has approximately 829,868 m of coastline, ranking 12th out of 39 coastal prefectures, whereas Mie Prefecture has approximately 1,140,150 m, ranking seventh, based on official statistics on coastal length published by the Ministry of the Environment. This reflects the characteristics and differences in coastal geographical conditions and marine resource endowments (Ministry of the Environment, 2016).

In the context of modernization and demographic change, local fish-based dietary patterns have been declining in consumption while maintaining certain social meanings in traditions or tourism, thereby providing a unique opportunity to gain insights into the relationship between dietary habits and cultural framing.

The questionnaire consisted of 18 items covering five main domains. The first was current fish consumption behaviors, including the frequency of fish intake and the use of locally sourced fish. The second was changes in fish utilization and fish-eating habits over the past decade, with particular attention to the influence of environmental and social structural transformations. The third involved perceptions of and emotional attitudes toward local cuisine, including a sense of identification and the degree of preference. The fourth concerned the importance of attitudes toward and perceptions of underutilized fish species. Finally, the fifth domain included a comparative variable, that is, respondents’ acceptance intentions regarding gene-edited food products as a form of food technology.

### 2.2 Measurement of variables

This study operationalized variables in accordance with the analytical framework of “habits– memory–framing.” Variable selection was grounded in prior research on the sociology of food, food-related memory studies, and cultural framing.

The independent variable was dietary habits, which distinguished between at-home and out-of-home dining habits. Existing studies have indicated clear situational differences among food practices, as different dining settings generate distinct patterns of interaction and experiential structures. Warde (2016) found that eating habits are embedded in specific social contexts, with at-home and out-of-home dining differing systematically in their modes of social organization and interactional logic. Research on consumption and food practices further indicates that dining at home is typically associated with intergenerational interactions and emotional exchanges, whereas out-of-home dining is more strongly shaped by market mechanisms and temporal constraints (Marshall, 1995). Therefore, distinguishing between changes in opportunities for seafood consumption in at-home versus out-of-home dining settings is crucial to identify how different dietary practices influence individuals’ psychological experiences.

The mediating variable was food-related memory. While existing research suggests that emotion plays a reinforcing role in memory formation, whereby stronger emotional experiences are more likely to be retained and influence subsequent cognitive processes (Schacter et al., 2007; Marshall, 1995), this study explicitly operationalized food-related memory as multidimensional subjective and emotional experiences evoked when consuming local cuisine. Specifically, it is defined and measured through self-reported feelings and associations of respondents during local dish consumption. Based on the survey design, this variable captures three core dimensions of food memory: (1) sensory and affective responses (e.g., feeling comfortable, appreciating flavor and freshness, and sensing seasonality), (2) socio-cultural and historical connections (e.g., perceiving regional uniqueness in ingredients or seasonings and sensing local history or festival-related specialness), and (3) interpersonal and nostalgic associations (e.g., evoking memories of family or the region and feeling gratitude toward producers). By synthesizing these multidimensional cognitive and emotional responses, we aimed to conceptualize how routine dietary habits are transformed into more stable psychological structures, thereby providing a measurable basis for food-related memory.

Cultural psychology research suggests that individuals' understanding of cultural change is grounded in personal experience and memory rather than being a mere reflection of objective facts. For example, Lizardo (2025) emphasized that cultural realities are constituted through individuals' subjective interpretations, while Schwartz (2012) argued that perceptions of social and value change can be captured through subjective evaluations. Reflecting on these debates and lessons learned from existing studies, we aimed to examine whether dietary habits influence individuals’ judgments about the continuity of local cuisine through the mediating role of food-related memory. The dependent variable was thus conceptualized as cultural perception, defined as whether individuals perceive that local cuisine has become “unavailable” or that opportunities to consume it have decreased.

Additionally, we integrated learning intention, frequency of fish consumption, co-residing generations, age, and place of residence as control variables, thereby enhancing the explanatory precision of the model estimation and minimizing potential confounding effects arising from demographic and experiential differences.

### 2.3 Data analysis

Data were analyzed using IBM SPSS Statistics 24.0. First, descriptive statistics were used to examine the basic relationships among the principal variables. Multiple linear regression models were employed to test the effects of dietary habits on food-related memory. Specifically, food-related memory was specified as the dependent variable, whereas at-home and out-of-home dining dietary habits were entered as key independent variables. Learning intention, frequency of fish consumption, co-residing generations, age, and place of residence were simultaneously included as control variables to identify the independent explanatory effects of dining contexts on food-related memory.

After testing the direct effects, a mediation model was constructed to examine the mediating role of food-related memory in the relationship between dietary habits and cultural perceptions. In this model, cultural perception was specified as the dependent variable, at-home and out-of-home dining dietary habits were treated as independent variables, and food-related memory was introduced as the mediating variable while controlling for the same set of demographic and individual characteristics included in the regression model. Mediation was assessed by comparing changes in the regression coefficients for cultural perception before and after the inclusion of food-related memory, in conjunction with testing the statistical significance of the indirect effects.

The indirect effects were evaluated using a Bootstrap resampling procedure with bias-corrected confidence intervals. When the confidence interval for the indirect effect did not include zero, the mediating role of food-related memory in the relationship between dietary habits and cultural perceptions was considered statistically significant. This approach does not rely on the assumption of a normal distribution and provides a more robust estimation of indirect effects.

## 3 Results

### 3.1 Descriptive statistics

A total of 840 valid questionnaires were collected, with an equal distribution of male and female respondents (50% each), resulting in a balanced gender composition. The survey targeted residents aged above the high school level living in the two prefectures. Data were collected between December 4 and 5, 2024, using an online questionnaire administered and distributed through an online survey company. A total of 840 valid responses were obtained, comprising 420 respondents each from Miyagi and Mie prefectures.

The distributions of age, occupation, and household composition are presented in Table 1.

**Table 1:** Sample characteristics (N = 840).

Variable	Category	N	%
Gender	Male	420	50.0
	Female	420	50.0
Age	29 years or younger	168	20.0
	30–49 years	336	40.0
	50 years or older	336	40.0
Occupation	Corporate employee	345	41.0
	Part-time worker	142	16.9
	Homemaker	113	13.5
	Student	54	6.4
	Unemployed	74	8.8
	Other	112	13.4
Household Composition	Living with spouse/partner	465	55.4
	Living with parents	220	26.2
	Living with children	281	33.5
	Living alone	147	17.5

The age structure was relatively evenly distributed across three groups: 20% were aged 29 years or younger, 40% were between 30 and 49 years, and 40% were aged 50 years or older, indicating a predominance of middle-aged and older respondents.

In terms of occupation, corporate employees constituted the largest group (41%), followed by part-time workers (16.9%), and homemakers (13.5%). Students and unemployed respondents together accounted for approximately 15%, whereas the remaining were civil servants, self-employed individuals, or those engaged in other occupations. Regarding household composition, 55.4% of respondents lived with a spouse or partner, 26.2% lived with their parents, 33.5% lived with their children, and 17.5% lived alone. Thus, the sample included a variety of household types, including nuclear families, multigenerational households, and single-person households.

Building on the description of sample characteristics, descriptive statistics were calculated for the key variables included in this study, as presented in Table 2. Regarding dietary practices, the mean value for seafood consumption in the at-home dining context was 2.92 (SD = 0.85), whereas in the out-of-home dining context, it was 2.87 (SD = 0.92). These two values are relatively close, suggesting that respondents perceived a similar level of opportunity to consume seafood at home and in out-of-home dining settings. In terms of cultural perception, the mean value for the perceived decline in opportunities to consume local cuisine was 3.45 (SD = 0.85), indicating that a certain proportion of respondents perceived a reduction in opportunities to consume local dishes in recent years. From the emotional dimension, the mean value for emotional memories associated with local cuisine was 0.24 (SD = 0.22). This variable was constructed by aggregating multiple items related to emotional experiences and was subsequently standardized, with values ranging from 0 to 1. The results suggested that respondents retained a certain level of food-related memory related to the local cuisine, although the overall level remained relatively moderate to low.

**Table 2:** Descriptive statistics of the key variables.

Variables	N	Minimum	Maximum	Mean	Standard Deviation
Seafood consumption in the at-home dining context	840	1	5	2.92	0.85
Seafood consumption in the out-of-home dining context	840	1	5	2.87	0.92
Perceived decline in opportunities for local cuisine consumption	840	1	5	3.45	0.85
Memories associated with the local cuisine	840	0.00	1.00	0.24	0.22

Overall, descriptive statistics provided a preliminary basis for understanding the relationships among seafood dietary practices, food-related memory, and cultural perception, laying the foundation for subsequent multiple regression and mediation analyses.

### 3.2 Multiple regression analysis

Two multiple linear regression models were constructed to examine the effects of different dietary contexts on food-related memory (Table 3). Model 1 introduced changes in the opportunities to consume seafood dishes in at-home dining settings as a key independent variable, whereas Model 2 introduced changes in the opportunities to consume seafood dishes in out-of-home dining settings. This analysis aimed to assess the direct effect of dietary habits on food-related memory from a single regression perspective, serving as a complementary step to the subsequent mediation analysis.

**Table 3:** Result of the multiple regression analysis.

Variables	Model1 B	SE	Beta	p	Model 2 B	SE	Beta	p
Constant	0.968	0.765	-	0.201	1.373	0.692	-	0.048
At-home dining	0.087	0.109	0.028	0.424	-	-	-	-
Out-of-home dining	-	-	-	-	−0.022	0.095	−0.088	0.815
Learning intention	1.028	0.079	0.424	<0.001	1.044	0.080	0.431	<0.001
Fish consumption frequency	−0.352	0.100	−0.124	<0.001	−0.381	0.093	−0.134	<0.001
Age	−0.042	0.060	−0.022	0.490	−0.050	0.062	−0.026	0.423
Prefecture	−0.416	0.166	−0.079	0.012	−0.461	0.166	−0.079	0.012
Co-residing generations	0.003	0.120	0.001	0.982	0.001	0.121	0.000	0.995
R^2^	0.240				0.239			
Adjusted R^2^	0.234				0.233			
F	40.920				40.792			
N	785				785			

In terms of the overall model fit, both models reached statistical significance (Model 1: F = 40.920; Model 2: F = 40.792; p < 0.001), indicating that the independent and control variables jointly provided meaningful explanatory power for food-related memory. The R^2^ values for Models 1 and 2 were 0.234 and 0.233, respectively, suggesting comparable explanatory capacities. Replacing the dietary habit indicator with control variables did not substantially alter the overall model fit. Regarding the focal independent variables, changes in opportunities to consume seafood dishes in at-home dining settings were not statistically significant in Model 1 (p = 0.424), and changes in opportunities to consume seafood dishes in out-of-home dining settings were likewise not significant in Model 2 (p = 0.815). Thus, after controlling for the relevant variables, neither type of dietary context had a significant direct effect on food-related memory. This suggests that, from the perspective of behavioral frequency or exposure alone, daily dietary habits are insufficient to directly explain the formation of food-related memory among individuals. Concerning control variables, learning intention demonstrated a significant positive effect in both models (Model 1: β = 0.424; Model 2: β = 0.431; p < 0.001), with the largest standardized coefficients among all predictors. Frequency of fish consumption showed a significant negative effect in both models (Model 1: β = −0.124; Model 2: β = −0.134; p < 0.001). Place of residence was also statistically significant in both models (p = 0.012), whereas age and co-residing generations did not reach statistical significance.

From the pattern of variable effects, the significant role of learning intention indicates that, compared with behavioral exposure or frequency, individuals’ cognitive motivation and cultural interest play a more critical role in the formation of food-related memory. This trend suggests that food-related memories are not passively accumulated through routine behavior but rather depend on the extent to which individuals actively engage with and interpret food-related cultural experiences. Thus, food-related memory is better understood as a psychological construct driven by cognitive engagement rather than a simple outcome of behavioral repetition. These results imply that the influence of dietary habits on food-related memory is not evident through a single direct pathway but instead operates within a more complex mechanism that cannot be captured by a simple linear relationship. This result also provides a basis for re-examining the relationships among the paths in the subsequent mediation analysis.

### 3.3 Mediation analysis

**Fig. 4:**
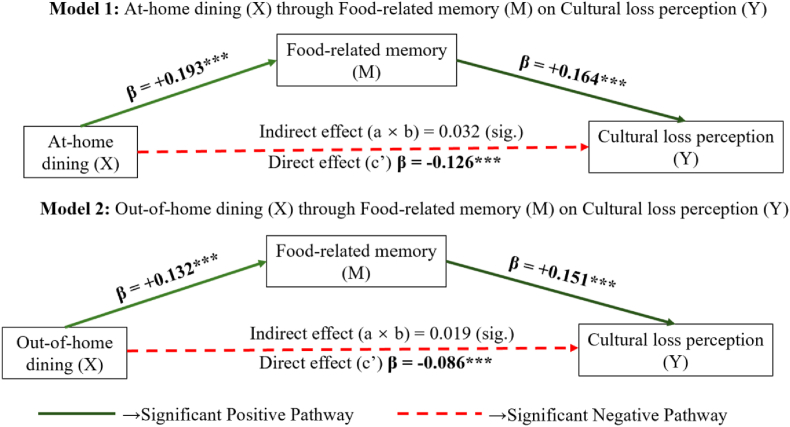
Path diagrams of the suppressive mediation models.

Building on this analysis, a mediation model was constructed to examine how daily seafood dietary habits influence the perception of cultural loss through food-related memory (Table 4). As shown in Fig. 4, our primary aim was to identify how Y (perception of cultural loss) is affected by X (at-home dining/out-of-home dining) or M (food-related memory). In principle, the mediation model allows for the identification of the overall mechanism linking variables, thereby providing a more comprehensive understanding of the role of dietary habits in the formation of cultural perceptions, unlike multiple regression, which captures relationships along a single direct pathway.

**Table 4:** Comparison of the mediating models for at-home and out-of-home dining seafood consumption.

Path	At-home dining (β)	p	Out-of-home dining (β)	p
X → M (a)	0.193	<0.001	0.132	<0.001
M → Y (b)	0.164	<0.001	0.151	<0.001
X → Y (direct, c’)	−0.126	<0.001	−0.086	0.015
Total effect (c)	−0.095	0.006	−0.066	0.061
Indirect effect (a × b)	0.032	Boot 95% CI [0.015, 0.051]	0.019	Boot 95% CI [0.006, 0.039]
R^2^ (Y model)	0.053		0.045	

(β represents the standardized coefficient).

First, in both at-home and out-of-home dining contexts, everyday seafood consumption habits were found to exert a significant positive effect on food-related memory (at-home dining: β = 0.193, p < 0.001; out-of-home dining: β = 0.132, p < 0.001). This finding contrasts with the non-significant relationship observed in the multiple regression analysis, suggesting that the effect of dietary habits on food-related memory does not manifest as a stable, independent relationship in a single-model specification but rather emerges within the broader mechanism of the overall analytical framework.

Second, food-related memory showed a significant positive effect on perceived cultural decline in both contexts (at-home dining: β = 0.164, p < 0.001; out-of-home dining: β = 0.151, p < 0.001). In other words, stronger emotional experiences are associated with a heightened perception that the local cuisine is disappearing. The indirect effect in the at-home dining context was 0.032, with a Bootstrap 95% confidence interval of [0.015, 0.051]; in the out-of-home dining context, the indirect effect was 0.019, with a Bootstrap 95% confidence interval of [0.006, 0.039]. As neither confidence interval included zero, food-related memory was regarded as playing a statistically significant mediating role in the relationship between everyday seafood consumption habits and perceived cultural decline in both contexts.

Examination of the direct effects further revealed that in the at-home dining seafood context, everyday seafood consumption habits continued to exert a significant direct effect on perceived cultural decline (β = −0.126, p < 0.001). Similarly, in the out-of-home dining context, the direct effect remained significant (β = −0.086, p = 0.015). These findings indicate a partial mediation structure: everyday seafood consumption habits influence perceived cultural decline, both directly and indirectly, through food-related memory. The direction of the direct effect suggests that more stable or frequent seafood consumption is associated with a lower direct perception of cultural decline, reflecting a behavioral buffering effect.

A comparison between the two contexts showed that the effect of seafood consumption habits on food-related memory was stronger in the at-home dining context than in the out-of-home dining context, and the magnitude of the indirect effect was likewise greater in the at-home dining setting. This suggests that the family remains a core arena for the construction of food-related memory and cultural identification related to the local cuisine. By contrast, the effect of food-related memory on perceived cultural decline differed only slightly across contexts, indicating that emotional mechanisms demonstrate relative contextual stability.

Overall, everyday seafood consumption habits simultaneously reduced perceived cultural decline through a negative direct pathway while reinforcing awareness of decline through a positive indirect pathway via food-related memory. Because the direct and indirect effects operated in opposite directions, the overall model exhibited a typical suppression-type mediation structure. The total effect was significant in the at-home dining context (β = −0.095, p = 0.006) and marginally significant in the out-of-home dining context (β = −0.066, p = 0.061), yet the coexistence of dual pathways was consistently supported across both dining contexts. Overall, the mediation analysis indicated that the effect of dietary habits on the perception of cultural loss was primarily realized through the psychological mechanism of food-related memory, thereby forming an integrated pathway of “habit–emotion–framing.”

### 3.4 Structural analysis of the decline in at-home and out-of-home dining dietary habits

#### 3.4.1 Reasons for the decline in the at-home dining context

While mediation analysis identifies the mechanism linking dietary habits, food-related memory, and cultural perception, it does not explain why seafood consumption is changing in the first place. Therefore, this study further examines the underlying reasons for changes in seafood dietary habits from a real-world perspective. Specifically, a statistical analysis of respondents who selected “slightly decreased” or “significantly decreased.” By categorizing and synthesizing the reported reasons, it is possible to identify the structural constraints underlying behavioral changes and provide the necessary contextual foundation for interpreting subsequent emotional mechanisms and cultural perceptions.

**Fig. 5:**
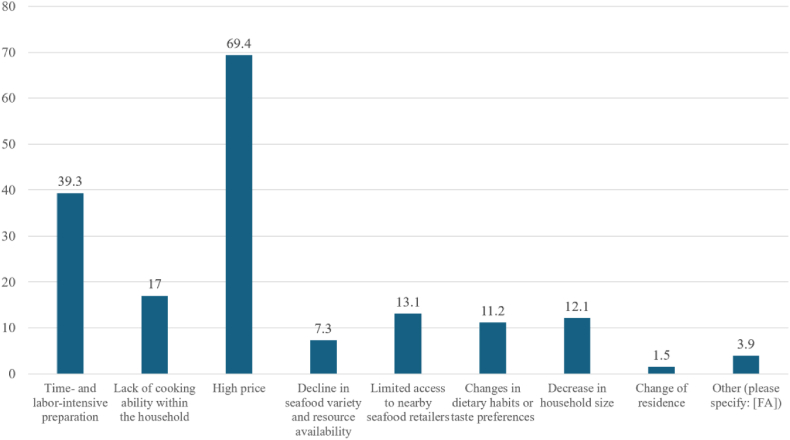
Composition of reasons for the decline in at-home dining seafood consumption habits.

As shown in Fig. 5, economic factors were the primary reason for the decline in seafood consumption in at-home dining settings. A total of 69.4% of respondents identified “high prices” as the main reason for reduced seafood consumption at home, indicating that cost pressures significantly constrain everyday dietary decisions. Beyond economic considerations, behavioral and time-related costs also play an important role. Specifically, 39.3% of the respondents reported that seafood preparation required considerable time and effort, 17.0% indicated a lack of cooking skills, 12.1% cited a decrease in the number of co-residing family members, and 1.5% reported changes in their place of residence. These findings suggest that changes in household structure and time allocation influence the maintenance of seafood-based dietary practices. In particular, within the context of shrinking household size and the increasing prevalence of dual-income families, cooking costs and household duties have become salient constraints on food choices.

Structural supply side factors also exert an influence. Approximately 7.3% of the respondents believed that seafood species or resource availability had declined, whereas 13.1% reported limited access to retail outlets where seafood could be conveniently purchased. These results indicate that market supply conditions and spatial accessibility shape household dietary behavior to a certain extent. Finally, 11.2% of respondents stated that seafood no longer aligns with their current dietary structure or taste preferences, reflecting an emerging shift in food culture. Overall, the decline in seafood consumption in the at-home dining context appears to reflect a multifactor structural pattern centered on economic and time constraints, compounded by changes in family structure and constraints in supply conditions.

#### 3.4.2 Reasons for the decline in the out-of-home dining context

**Fig. 6:**
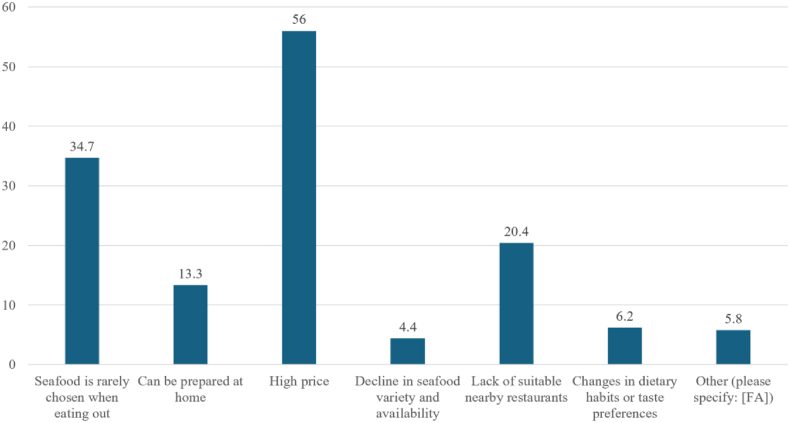
Composition of reasons for the decline in seafood consumption habits in the out-of-home dining context.

As shown in Fig. 6, economic factors are prominent in the out-of-home dining context. A total of 56% of respondents identified “high prices” as the primary reason for reducing their selection of seafood when dining out, indicating that cost considerations continue to play a decisive role in consumption settings. Market supply and spatial accessibility factors are more salient in the out-of-home dining context than in the at-home dining context. Specifically, 20.4% of the respondents reported a lack of suitable restaurants nearby that offer seafood options, whereas 4.4% perceived a decline in the variety or availability of seafood. These findings suggest that, in out-of-home dining situations, consumer choices are considerably constrained by the market supply structure.

In addition, 34.7% of the respondents indicated that they generally chose seafood less frequently when dining out, and 13.3% reported that seafood needs could already be satisfied through home cooking. These results reflect preference differentials in external dining contexts and suggest that seafood occupies a relatively marginal position in out-of-home dining consumption patterns. Changes in dietary preferences were also found to exert some influence, with 6.2% of respondents stating that seafood no longer aligned with their current dietary structure or taste preferences.

Overall, the decline in seafood consumption in the out-of-home dining context more clearly reflects a “market-choice logic,” whereby price factors and supply conditions jointly shape consumer decision-making.

#### 3.4.3 Comparison of the two contexts and mechanism analysis

A comparative analysis revealed that although economic factors dominated in both at-home and out-of-home dining contexts, the remaining structural determinants differed markedly. In the at-home dining setting, behavioral and time-related costs, along with changes in household structure, were more prominent, reflecting transformations in everyday life arrangements and the division of domestic labor. By contrast, in the out-of-home dining context, market supply conditions and consumption preference structures were more salient, highlighting the constraints imposed by price and accessibility within consumer environments.

Therefore, the decline in seafood consumption habits cannot be attributed to a single factor; rather, it results from the combined effects of rising economic costs, shifts in supply conditions, lifestyle transformations, and adjustments in dietary preferences. These structural realities constitute the foundation of behavioral change and provide contextual grounding for the tension observed in the mediation model between behavioral buffering effects and emotional mechanisms. Alternatively, a decline in behavioral frequency at the level of everyday practice may weaken individuals’ direct perception of “cultural decline.” Another explanation is that food-related memory continues to reinforce awareness of the vulnerability of local cuisine through psychological processes, thereby generating a complex suppression-type mediation structure.

## 4 Discussion

### 4.1 Interpretation of the findings and theoretical implications

This study examined seafood consumption habits to investigate the relationships among daily dietary habits, food-related memory, and the perception of the decline of local cuisine. Overall, the results indicate that seafood consumption habits in both at-home and out-of-home dining influence perceptions of cultural decline through food-related memory. However, this process is not a simple linear relationship but reflects a complex interplay between behavioral and emotional mechanisms. Multiple regression analyses showed that after controlling for relevant covariates, variations in seafood consumption opportunities in at-home and out-of-home dining do not exert significant direct effects on food-related memory, with learning intention demonstrating the most stable and pronounced positive effect. These findings suggest that food-related memory related to local cuisine is not solely determined by the frequency of consumption or exposure opportunities but is also influenced by individual cognitive motivation and cultural interests. Alternatively, food-related memory functions more as a psychological construct than a mere behavioral outcome. Prior studies have noted that food serves not only as a daily consumption object but also as a key component of personal memory and cultural identity. Individuals with higher interest or active learning intention toward a particular dietary culture are more likely to form memory connections through lived experiences (Holtzman, 2006; Sutton, 2001). Accordingly, our findings indicate that the formation of food-related memory depends not only on routine experience but also on individual motivation for cultural participation.

Further mediation analyses reveal the mechanisms linking dietary habits and perceptions of cultural decline. Seafood consumption habits in both at-home and out-of-home dining significantly enhance food-related memory, which in turn reinforces individuals’ perceptions of reduced opportunities to consume local dishes. This suggests that daily dietary habits remain a crucial pathway for sustaining experiential engagement with local food culture, allowing repeated behaviors to accumulate food-related memories and influence cognition. As Warde (2016) emphasized, daily eating habits not only shape consumption patterns but also construct cultural meanings through long-term lived experiences. Food experiences connected to locality and identity serve as references for understanding cultural changes (Mak et al., 2012a). Consequently, when the frequency of local cuisine consumption in daily life declines, individuals with stronger food-related memory are more likely to perceive such changes. Notably, this study found that the direct effect of seafood consumption habits on the perception of cultural decline is negative, while the indirect effect through food-related memory is positive, forming a suppressing mediation structure. This indicates that dietary habits simultaneously exert opposing influences on cultural framing: on one hand, sustained consumption habits reduce direct awareness of cultural decline at the behavioral level; alternatively, these habits amplify sensitivity to cultural change through accumulated memory mechanisms. Therefore, dietary habits can buffer and heighten awareness of cultural decline. From the perspective of food heritage, local dishes as cultural resources are often perceived as “disappearing” when their presence in daily life diminishes (Bessière, 2013), which aligns with the findings of this study.

Moreover, an analysis of the reasons behind the decline in seafood consumption habits reveals the practical bases of behavioral changes. In the household context, price and cooking time constraints are the main influencing factors, while structural family changes and the accelerated pace of life weaken the continuity of household dietary habits. By contrast, in the out-of-home dining context, market supply conditions and spatial accessibility play a more prominent role, indicating that consumption choices are largely constrained by market structures. As Johnston and Baumann (2010) noted, contemporary eating behaviors are increasingly influenced by market conditions and consumption environments. Therefore, the results suggest that changes in dietary habits are not solely driven by cultural factors but also reflect the combined effects of socioeconomic conditions and lifestyle transformations.

In summary, this study demonstrates that the perception of cultural decline is shaped by a complex interplay of individual motivation, structural realities, and memory mechanisms, this study proposes a “habit–memory–framing” framework that explains how changing dietary habits reshape food-related memory and, ultimately, frame perceptions of cultural decline. First, the formation of food-related memory is significantly driven by active learning intentions and cultural interests rather than merely by the frequency of consumption. Second, the actual decline in daily dietary habits is fundamentally constrained by structural barriers, such as high prices, cooking time constraints, and limited market accessibility across both at-home and out-of-home dining contexts at a practical level. Finally, under these structural constraints, food-related memory serves as a suppressing mediator. While the maintenance of dietary habits buffers the direct awareness of cultural loss at the behavioral level, it simultaneously amplifies cognitive sensitivity to this decline through accumulated memory. By integrating structural realities (e.g., economic and market constraints) with individual emotional and cognitive experiences, this study provides a comprehensive framework for understanding how local food heritage is experienced and processed during periods of cultural transformation. The suppression effect also indicates that behavioral experiences and memory-based framing can exert divergent, even opposing, effects when analyzing changes in food culture. Consequently, shifts in food culture are not merely the outcome of changing consumption patterns but are also closely associated with individual emotional experiences and modes of cultural framing.

### 4.2 Limitations and future research

First, this study is based on cross-sectional survey data, which limits the ability to establish causal relationships between variables. Future research could employ longitudinal data to further validate the underlying mechanisms. Second, from a methodological perspective, this study employs a mediation model to examine the relationships among the variables. This approach relies on assumptions such as linearity and may not fully capture the more complex and dynamic interactions among dietary habits, food-related memory, and cultural framing. Food-related memory, as a subjective psychological construct, is primarily measured using self-reported questionnaires that may not fully reflect its multidimensional nature. Furthermore, the explanatory power of the model is relatively limited (low R^2^), suggesting that other important factors not included in this study may influence cultural framing. Future research could incorporate additional variables or adopt mixed-methods approaches to enhance the explanatory power and robustness of the analysis.

## 5 Conclusion

The results indicate that seafood consumption habits exert both direct and indirect effects, mediated by food-related memory, on the perception of a decline in seafood culture, with the two pathways operating in opposite directions. This finding suggests that individuals’ perceptions of changes in food culture are not determined by a single factor, but emerge from the combined influence of habitual practices and emotional experiences. Furthermore, this study highlights the critical role of food-related memory in linking dietary habits with cultural perception, underscoring the need to integrate behavioral and emotional mechanisms when analyzing changes in local food culture, rather than focusing solely on consumption frequency or product availability. This study applied an analytical framework of “dietary habits–food-related memory–perception of cultural decline” to examine the mechanisms through which at-home and out-of-home dining influence changes in local food culture.

These findings have both policy and practical implications. First, structural measures are required to alleviate practical constraints on seafood consumption, such as optimizing distribution systems, stabilizing prices, and improving product accessibility, thereby lowering barriers to daily seafood consumption and the accompanying local culture. Second, differentiated strategies should be adopted for both at-home and out-of-home dining. In the household context, promoting convenient cooking methods and dietary education can reduce time and skill costs and support the maintenance of seafood consumption habits. In the context of out-of-home dining, encouraging the catering industry to increase the availability of seafood dishes can enhance both visibility and choice. Finally, emotional and cultural guidance should be strengthened through food culture dissemination, experiential activities, and educational programs, fostering public emotional attachment to local cuisine and raising awareness of changes in food culture.

Overall, by integrating dietary habits with food-related memory, this study reveals the multi pathway mechanisms underlying changes in local food culture, providing new empirical evidence for understanding the evolution of food culture and offering actionable insights for policy-making and practical interventions.

## **Funding**

The work has been financially supported by the JSPS KAKENHI [JP22H03852; JP23H01584; JP23H03605]; JST, COI-NEXT [JPMJPF2110]; and the Environment Research and Technology Development Fund of the Environmental Restoration and Conservation Agency provided by Ministry of the Environment of Japan [JPMEERF20241M03].

## Declaration of competing interest

This manuscript has not been published or presented elsewhere in part or in entirety and is not under consideration by another journal. We have read and understood your journal's policies, and we believe that neither the manuscript nor the study violates any of these. There are no conflicts of interest to declare.

## Data Availability

Data will be made available on request.
